# Cord Blood Platelet Lysate-Loaded Thermo-Sensitive Hydrogels for Potential Treatment of Chronic Skin Wounds

**DOI:** 10.3390/pharmaceutics16111438

**Published:** 2024-11-11

**Authors:** Arianna Grivet-Brancot, Marianna Buscemi, Gianluca Ciardelli, Simona Bronco, Susanna Sartori, Claudio Cassino, Tamer Al Kayal, Paola Losi, Giorgio Soldani, Monica Boffito

**Affiliations:** 1 Institute for Chemical-Physical Processes, National Research Council, 56124 Pisa, Italy; arianna.grivet@polito.it (A.G.-B.); simona.bronco@pi.ipcf.cnr.it (S.B.); 2 Department of Mechanical and Aerospace Engineering, Politecnico di Torino, 10129 Torino, Italy; susanna.sartori@polito.it; 3 Institute of Clinical Physiology, National Research Council, Massa, 56124 Pisa, Italy; maribuscemi@libero.it (M.B.); tamer.alkayal@ifc.cnr.it (T.A.K.); paola.losi@cnr.it (P.L.); giorgio.soldani@ifc.cnr.it (G.S.); 4 Department of Science and Technological Innovation, Università del Piemonte Orientale, 15121 Alessandria, Italy; claudio.cassino@uniupo.it

**Keywords:** platelet lysate, hydrogels, chronic skin wounds

## Abstract

Background/Objectives: Chronic skin wounds (CSWs) are a worldwide healthcare problem with relevant impacts on both patients and healthcare systems. In this context, innovative treatments are needed to improve tissue repair and patient recovery and quality of life. Cord blood platelet lysate (CB-PL) holds great promise in CSW treatment thanks to its high growth factors and signal molecule content. In this work, thermo-sensitive hydrogels based on an amphiphilic poly(ether urethane) (PEU) were developed as CB-PL carriers for CSW treatment. Methods: A Poloxamer 407^®^-based PEU was solubilized in aqueous medium (10 and 15% *w*/*v*) and added with CB-PL at a final concentration of 20% *v*/*v*. Hydrogels were characterized for their gelation potential, rheological properties, and swelling/dissolution behavior in a watery environment. CB-PL release was also tested, and the bioactivity of released CB-PL was evaluated through cell viability, proliferation, and migration assays. Results: PEU aqueous solutions with concentrations in the range 10–15% *w*/*v* exhibited quick (within a few minutes) sol-to-gel transition at around 30–37 °C and rheological properties modulated by the PEU concentration. Moreover, CB-PL loading within the gels did not affect the overall gel properties. Stability in aqueous media was dependent on the PEU concentration, and payload release was completed between 7 and 14 days depending on the polymer content. The CB-PL-loaded hydrogels also showed biocompatibility and released CB-PL induced keratinocyte migration and proliferation, with scratch wound recovery similar to the positive control (i.e., CB-PL alone). Conclusions: The developed hydrogels represent promising tools for CSW treatment, with tunable gelation properties and residence time and the ability to encapsulate and deliver active biomolecules with sustained and controlled kinetics.

## 1. Introduction

Wound healing is an extremely complex and dynamic process representing an important challenge in modern medicine. Indeed, it requires the integration of various biological and molecular events in several phases, involving different cytokines and growth factors (GFs) that act on the different cellular types involved in the wound healing cascade [1,2]. Chronic skin wounds (CSWs), lesions usually derived from diabetes or ischemia that do not heal spontaneously and do not respond to standard treatments, are even more difficult to address. They represent a severe problem, involving approx. 40 million patients worldwide [3] with a high cost for healthcare systems (estimated USD 26 billion by the end of 2023 [4]) due to the relevant need for prolonged medical assistance and frequent hospitalization. Furthermore, CSWs also lead to severe consequences on patients’ social and mental health due to prolonged healing times, reduced quality of life, and impaired mobility and workability. The CSW gold standard treatment encompasses wound cleaning, debridement, the application of dressings, infection control and management, and nutritional support [5]. Although traditional dressings have significantly evolved in recent decades to address the challenges raised by these pathologies, innovative treatments are still required to improve clinical performances in this field, as thoroughly surveyed in recent reviews [6,7,8,9]. In detail, traditional dressings lack the ability to maintain a proper moist environment, which is needed for wound healing; require frequent replacement; often stick to the wound bed, causing secondary damages and decreasing patient compliance; and do not show any bioactive or smart features that make them able to take part effectively and actively in the wound healing cascade.

Several types of advanced wound dressings have been developed, with some examples already available on the market [10,11,12]. Devices containing active agents, for example, to prevent or contain bacterial infection through silver ions [13,14,15] or antibacterial drugs release [16,17], to enhance wound closure by exploiting conductive properties [18], or to actively stimulate the healing process using specific GFs, such as the vascular endothelial growth factor (VEGF) [19] or the basic fibroblast growth factor (bFGF) [20,21], have already been extensively studied. However, even the only FDA-approved treatment based on platelet-derived growth factor (PDGF) [22] reported controversial results in clinical trials, and thus, its clinical application is still limited [23]. In general, the direct use of GFs in the wound bed is not highly effective because of rapid diffusion followed by enzymatic digestion or inactivation [24]; moreover, the use of a single GF can be limiting for processes as complex as wound healing and tissue regeneration, which require multiple signals to be properly triggered [25]. The use of concentrated platelet derivatives containing several bioactive molecules, such as GFs and other mediators (e.g., cytokines and chemokines), could represent a synergic stimulus more similar to the physiological one since platelets play a fundamental role in tissue repair [26]. Platelet lysate (PL), obtained through a freeze-thawing process from platelet-rich plasma, could be particularly interesting in this sense since it contains immediately available GFs and cytokine cocktails. Platelet-derived growth factor (PDGF) is the most representative growth factor present in platelet lysate, playing a pivotal role in promoting and accelerating wound healing, as demonstrated by many authors that incorporated it in hydrogel formulations [27,28]. Additional GFs present in PL are vascular endothelial growth factor (VEGF), fibroblast growth factor (FGF), transforming growth factor-beta (TGF-β), and insulin-like growth factor 1 (IGF-1) [29]. Hence, compared to the use of single GFs, PL holds great advantage due to its multi-component nature. For instance, Liao et al. demonstrated that PL at a 0.5% concentration was better than 20 ng/mL of bFGF and 10% fetal bovine serum (FBS) in promoting dental pulp stem cell survival within 24 h [30].

Platelet derivatives are often of autologous origin, but this can be a limiting factor when patients present previous pathologies or an advanced age. Furthermore, the quality of the collected PL varies among patients, and the need to collect a high volume of blood causes additional patient discomfort and health-related issues [29].

A possible alternative is represented by umbilical cord blood platelet lysate (CB-PL), which exhibits a GF content 4- to 5-folds higher than PL derived from adult blood and an immunology immaturity that makes it safer for clinical use. Moreover, its availability is guaranteed by public cord blood banks, since about 80% of the cord blood units stored are not suitable for transfusion due to an insufficient cellular count, making it an inexpensive source for PL production [31]. CB-PL and other cord blood derivatives have already shown potential for wound healing in several studies [32,33], especially regarding diabetic ulcers [34,35]. Despite the good properties of CB-PL, its direct application on the wound bed can limit its efficacy over time due to the rapid metabolization and diffusion of bioactive agents, as previously discussed for single GFs. For this reason, drug delivery systems able to guarantee a sustained release over a reasonable period of time while preserving the bioactivity of the payload could represent a more sustainable solution to daily injections and waste of material [36,37,38,39].

Hydrogels are ideal candidates in this sense and have already been used for these purposes in several studies [10,11]. For instance, Cahhal et al. recently demonstrated that PL loading provided poly(ethylene glycol)-based hydrogels with improved bioactivity and the capability to attract stem cells in vitro [40]. In other works, the authors reported that PL encapsulation into hydrogels improved the in vitro viability, proliferation, and differentiation potential of mesenchymal stem cells [41,42,43]. In the same year, hyaluronic acid (HA)-based microgels were developed for PL delivery and loaded into HA bulk hydrogels to improve the proliferation of human bone-marrow-derived mesenchymal stem cells [44]. Hydrogels have also been used as wound dressing for a long time because they are able to provide a moist environment and can be easily modified to obtain useful properties, such as adhesiveness, enhanced fluid absorbance, or anti-inflammatory and antibacterial properties [45,46]. For instance, PL-loaded keratin hydrogels were successfully developed with tunable physico-chemical properties and release kinetics depending on the keratin and PL contents within the formulation [47]. Notodihardjo and co-workers also performed a comparative study among a gelatin hydrogel sheet, direct PL administration, and their combination through impregnation, evidencing that the PL-impregnated hydrogel accelerated granulation tissue and capillary formation compared to both the hydrogel alone and PL alone [38]. Recently, Jin et al. incorporated PL into gelatin methacryloyl aqueous solutions, and the resulting photo-cured gels showed optimal biocompatibility and favorable effects on essential cells for wound healing [39]. Gelatin was also combined with chitosan to develop PL containing hydrogels crosslinked via β-glycerophosphate and glyoxal [48]. The authors reported that the PL-incorporated hydrogel significantly promoted fibroblast proliferation and human umbilical vein endothelial cell migration and angiogenesis in vivo. Similarly, Lim and colleagues reported improved wound healing, collagen deposition, and neovascularization in the wound sites of rats treated with a thermo-sensitive PL-loaded chitosan hydrogel compared to animals implanted with the pristine hydrogel [36]. In the same year, Bernal-Chavéz et al. exploited a Pluronic^®^ F127 thermosensitive hydrogel as a carrier of PL-loaded poly(lactic-co-glycolic)acid (PLGA) nanoparticles and tested the potential of this hybrid formulation for the treatment of wounds. The authors reported that the semi-occlusive environment created by the hydrogel upon application, coupled with the released PL from the nanoparticles, effectively promoted tissue regeneration [37]. The use of materials of synthetic origin, like Pluronic^®^ F127, for hydrogel preparation provides clear advantages in terms of repeatability and availability. However, Pluronic^®^ F127-based hydrogels are well known in the scientific community for their drawbacks concerning their rapid disintegration in bodily fluids and inadequate mechanical and bioadhesive characteristics [49], resulting in fast erosion in watery environments and, consequently, bursting and the quick delivery of potentially encapsulated payloads (e.g., Bernal-Chavéz et al. reported a complete release of freely encapsulated PL in a Pluronic^®^ F127 hydrogel at 20% *w*/*v* within 12 h, which was extended to 24 h with PL loading into PLGA particles [37]). In this context and with the aim to overcome the aforementioned limitations of Pluronic^®^ F127-based hydrogels while keeping the advantages typical of synthetic polymers, our group has been working on the synthesis of amphiphilic biodegradable and biocompatible poly(urethane)s starting from commercial Poloxamer^®^ 407 and their use to engineer thermo-sensitive hydrogels able to undergo a sol-to-gel transition with increasing temperature [50,51,52]. Aqueous solutions of these materials provide improved properties compared to similar Poloxamer^®^ 407-based systems, such as faster gelation (e.g., a gelation time of 5 min vs. 30 min at an 18% *w*/*v* concentration and a temperature of 37 °C), the capability to incur in the sol–gel transition at lower concentrations (a critical gelation concentration of 6% *w*/*v* vs. 18% *w*/*v*), greater mechanical strength (a storage modulus of around 40 kPa vs. 10 kPa at 37 °C and a 20% *w*/*v* concentration), superior stability in a physiological environment (at a 20% *w*/*v* concentration and 37 °C, stability for up to 28 days vs. 5 days), and the ability to better and more firmly maintain their shape [50]. These systems are also injectable, thus making them suitable for direct application even in irregular wound sites, and they have shown great potential as drug delivery systems [51,52,53,54].

In this work, we exploited these highly promising systems to incapsulate CB-PL to engineer a new candidate wound patch able to concurrently provide (I) an acceptable residence time in the wound bed, and (II) the controlled release of CB-PL over time, modulated by the poly(urethane) solution concentration, with the correct preservation of in vitro bioactivity. In detail, an amine-bearing poly(ether urethane) (PEU) was first synthesized using Poloxamer^®^ 407 (also commercially available as Pluronic^®^ F127), 1,6-hexamethylene diisocyanate, and N-Boc serinol as building blocks. Upon chemical characterization, the poly(urethane) was used to engineer thermo-sensitive hydrogels at two different polymeric concentrations (i.e., 10 and 15% *w*/*v*), which were later loaded with CB-PL at a 20% *v*/*v* concentration. The hydrogels were thoroughly physico-chemically characterized to assess their gelation properties and stability in an aqueous environment and evaluate the potential CB-PL contribution to these features. CB-PL release from the formulations was also tested, and the bioactivity of released CB-PL was evaluated through cell viability, proliferation, and migration assays using L929 murine fibroblasts and HaCat human keratinocytes, highlighting the great potential of the developed formulations for further investigation in the wound healing field.

## 2. Materials and Methods

### 2.1. Materials

Poloxamer^®^ 407 (P407, $\bar{M_{n}}$ 12,600 Da, 70% poly(ethylene oxide) (PEO) content), 1,6-hexamethylene diisocyanate (HDI), dibutyltin dilaurate (DBTDL), and N-Boc serinol were purchased from Merck Life Science, Milano, Italy. Before the poly(urethane) synthesis, P407 was anhydrified under vacuum at 100 °C for 8 h and then kept at 40 °C until use. HDI was distilled under vacuum to eliminate stabilizers and water residues, while N-Boc serinol was stored at room temperature and reduced pressure. Analytical grade solvents were purchased from Carlo Erba Reagents, Cornaredo, Italy. Before being used in the synthesis process, 1,2-dichloroethane (DCE) was anhydrified under N_2_ flow over molecular sieves (3 Å, Merck Life Science, Milano, Italy) previously activated at 120 °C. The bicinchoninic acid assay (BCA kit) and ELISA test were purchased from Merck Life Science, Milano, Italy and used as received.

### 2.2. Poly(urethane) Synthesis

The synthesis of the PEU named NHP407 was carried out following a previously published two-step reaction protocol [51]. Briefly, P407 was solubilized in anhydrous DCE at a 20% *w*/*v* concentration in inert atmosphere (continuous N_2_ flow). After temperature equilibration at 80 °C, HDI was added at a 2:1 molar ratio with respect to P407, and the reaction was carried out for 150 min in the presence of DBTDL (0.1% *w*/*w* with respect to P407) as catalyst. Then, temperature was brought to 60 °C, N-Boc serinol was added (1:1 molar ratio with respect to P407; solubilization at 3% *w*/*v* concentration in anhydrous DCE), and the second step was carried out for 90 min. The reaction was finally stopped by cooling down the reaction mixture to room temperature and passivating the unreacted isocyanate groups with methanol. The PEU was then collected by precipitation in petroleum ether (4:1 volume ratio with respect to the DCE used) and purified by dissolution in DCE (35% *w*/*v*) and subsequent precipitation in a solution (5:1 volume ratio with respect to DCE) of diethyl ether:methanol (98:2 volume ratio). The solution was centrifuged (6000 rpm, 20 min, 0 °C, MIKRO 220R—Hettich, Kirchlengern, Germany), and the collected polymer was dried overnight under a fume hood and stored at 4 °C under vacuum.

### 2.3. NHP407 Deprotection (Boc-Protected Amine Exposure)

The Boc-deprotection reaction was performed as previously described [51], and the resulting deprotected PEU was identified with the acronym SHP407. In brief, NHP407 (10 g) was dissolved and stirred for 120 min in 225 mL of chloroform at room temperature under N_2_ flux. Then, 25 mL of trifluoroacetic acid (TFA) was added, and the solution was stirred for 60 min. The excess TFA and chloroform were eliminated by rotary evaporation (Rotavapor^®^ Labortechnik AG, BUCHI Italia, Cornaredo, Italy). Then, to completely evaporate TFA traces, chloroform (10% *w*/*v*) was added to the concentrated solution and evaporated again under vacuum. This procedure was repeated twice. Lastly, distilled water was added at a 5% *w*/*v* concentration, and the solution was stirred overnight at 4 °C. Dialysis (cellulose membrane cut-off 12–14 kDa, Sigma Aldrich, Milano, Italy) was performed against demineralized water at 4 °C for 2 days (with complete dialysis medium refresh three times/day) and the dialyzed solution was finally lyophilized (Alpha 2-4 LSC, Martin Christ, Osterode am Harz, Germany). The final material was stored at 4 °C under vacuum until use.

### 2.4. PEU Chemical Characterization

#### 2.4.1. Attenuated Total Reflectance Fourier Transform Infrared (ATR-FTIR) Spectroscopy

Attenuated Total Reflectance Fourier Transform Infrared (ATR-FTIR) spectra were registered for P407, NHP407, and SHP407 to verify the success of the synthesis and deprotection protocols (i.e., the formation of urethane bonds and absence of PEU degradation during acidic treatment with TFA/chloroform). Analyses were performed at room temperature in the spectral range of 4000–600 cm^−1^ with a Perkin Elmer (Waltham, MA, USA) Spectrum 100 equipped with an ATR accessory (Perkin Elmer UATR KRS5, Waltham, MA, USA) with a diamond crystal. Each spectrum was obtained as the average of 32 scans (resolution 4 cm^−1^) and analyzed using the Perkin Elmer Spectrum 10 software.

#### 2.4.2. Size Exclusion Chromatography (SEC)

Number Average and Weight Average molecular weights ($\bar{M_{n}}$ and $\bar{M_{w}}$) and polydispersity index ($D=\bar{M_{w}}$/$\bar{M_{n}}$) of NHP407 and SHP407 were estimated by Size Exclusion Chromatography (SEC, Agilent Technologies 1200 Series, Santa Clara, CA, USA). The instrument was equipped with a Refractive Index Detector (RID) and two Styragel columns (HR1 and HR4) (Waters, Drinagh, Ireland) conditioned at 55 °C. N,N-Dimethylformamide (DMF, CHROMASOLV^®^ Plus, HPLC grade, 99.8%, Carlo Erba, Cornaredo, Italy) with lithium bromide (LiBr, 0.1% *w*/*v*, Sigma Aldrich, Milano, Italy) was used as eluent at a flow rate of 0.5 mL/min. $\bar{M_{n}}$ and $\bar{M_{w}}$ were determined by the B.04.03 Agilent ChemStation Software, referring to a calibration curve based on PEO standards (Peak molecular weight, $M_{p}$, range 982–205,500 Da). To perform the analysis, samples were dissolved in the previously described DMF/LiBr (2 mg/mL) mobile phase and filtered through a 0.45 μm syringe filter (polytetrafluoroethylene, Whatman, Maidstone, UK).

#### 2.4.3. Proton Nuclear Magnetic Resonance (^1^H NMR) Spectroscopy

Proton Nuclear Magnetic Resonance (^1^H NMR) spectroscopic analyses were conducted on NHP407 and SHP407 to assess the success of PEU synthesis and the absence of chemical degradation induced by the deprotection reaction, as well as to prove the successful cleavage of Boc-caging groups. Samples were prepared by solubilizing the polymer in deuterium oxide (D_2_O, 99.8%, Merck Life Sciences, Milano, Italy) and analyzed using an Avance III Bruker instrument equipped with an 11.74 T superconducting magnet (500 MHz 1H Larmor frequency) and a Bruker BBFO direct probe. The spectra were recorded at 25 °C and resulted from 12 scans with a 10 s relaxation time. Spectra were referred to the D_2_O peak at 4.675 ppm and analyzed using the MestReNova software (version 6.0.2-5475Mestrelab Research, S.L, Santiago de Compostela, Spain).

### 2.5. Design and Characterization of SHP407-Based Hydrogels

#### 2.5.1. Hydrogel Preparation

Sol–gel systems based on SHP407 were prepared by solubilizing the PEU at two different concentrations (10% *w*/*v* and 15% *w*/*v*) in phosphate-buffered saline (PBS, pH 7.4, Merck Life Science, Milano, Italy) in Bijou sample containers (Carlo Erba Reagents, Cornaredo, Italy, inner diameter 17 mm). The hydrogel composition was defined according to previous characterizations on analogous systems [50,51] to guarantee their thermo-sensitive response and the achievement of a gel state in physiological conditions. Polymer solubilization was ensured by keeping the samples at 4 °C overnight to avoid gelation. The obtained hydrogels will be referred to as SHP407 10% *w*/*v* and SHP407 15% *w*/*v* depending on the PEU concentration (i.e., 10 and 15% *w*/*v*, respectively).

#### 2.5.2. Tube Inverting Test

The tube inverting test was performed to estimate the temperature at which the sol-to-gel transition of the samples occurs, and the time required for the sol-to-gel transition to be completed in physiological conditions (i.e., 37 °C). To estimate the Lower Critical Gelation Temperature (LCGT), a controlled temperature increase was performed from 4 °C to 45 °C at 1 °C/step, maintaining the temperature for 5 min and inverting the vial for 30 s during each step. By observing the inverted vial, if a flow was still present, the sample was considered as a “flow liquid sol”, while in the absence of flow along the vial walls, the sample was defined as a “no flow solid gel”. Similarly, the gelation time at physiological temperature was determined by incubating the samples at 37 °C (IF 75, Memmert, Schwabach, Germany) and observing their state for 30 s at 1 min intervals until all samples could be classified as “no flow solid gel” as previously described. Visual inspections were independently performed by 3 different operators.

#### 2.5.3. Rheological Characterization

The rheological properties of the sol–gel systems were determined with a stress-controlled rheometer (MCR302, Anton Paar GmbH, Graz, Austria) by performing the measurements (i.e., strain sweep test, frequency sweep test, and temperature ramp test) with a 25 mm parallel plate geometry and by controlling the temperature with a Peltier system. Each sample was prepared as previously described and poured on the lower plate of the rheometer in the sol phase at 0 °C, heated at the testing temperature, and maintained for 15 min to reach stable thermal conditions. Strain sweep tests were conducted to define the linear viscoelastic (LVE) region and provide information about sample resistance to applied deformation. Analyses were performed at 37 °C at a frequency of 10 Hz with variable applied deformation (amplitude strain γ, range 0.01–500%). Hydrogel temperature-dependent gelation kinetics was then investigated by frequency sweep tests within the determined LVE region. The tests were conducted at 25 and 37 °C, applying a constant deformation and a variable angular frequency (range 0.1–100 rad/s). Temperature ramp tests allowed us to study the hydrogel viscosity (η) trend as a function of temperature variation. Tests were conducted at constant shear rate (0.1 Hz) and within the temperature range from 0 °C to 40 °C (2 °C/min).

### 2.6. CB-PL Loading into SHP407 Hydrogels and Characterization

#### 2.6.1. CB-PL Manufacturing

CB-PL was prepared from cord blood (CB) units collected in plastic bags (JMS, Hayward, CA, USA) containing citrate-phosphate-dextrose-adenine-1 anticoagulant according to validated standard operation procedures. The units were processed for the preparation of platelet-enriched-plasma within 48 hours from collection. Only CB units with total nucleated cells <1.5 × 10^9^ and platelets >200 × 10^6^ were enrolled in this study to obtain a target mean platelet concentration of 1 × 10^9^/L (range of 0.8–1.2 × 10^9^/L according to the platelet concentration defined by the Italian Society of Transfusion Medicine for platelet gel obtained from adult blood) and a target mean volume of 10 mL (range of 5–15 mL). The CB units were finally cryopreserved in a −80 °C mechanical freezer. CB-PL was obtained by thawing the platelet concentrate at 37 °C.

#### 2.6.2. Ethic Statement

The Italian Cord Blood Network has a program to collect whole blood and prepare donor-derived platelet concentrate and has been accredited by the Regional Health Authority. Approval from an institutional review board or ethics committee was thus not necessary. However, written informed consent was obtained from all the parents, and they were informed that the cord blood units not eligible for unrelated transplantation were used for research.

#### 2.6.3. Preparation of CB-PL-Loaded Hydrogels

CB-PL was loaded into the SHP407-based hydrogels at a final concentration of 20% *v*/*v*. The procedure used to obtain the systems was adapted from [52] to facilitate solubilization and a homogeneous dispersion of CB-PL in the final solution. In brief, SHP407 powders were first dissolved in a volume of PBS equal to 80% of the final one required to obtain the defined hydrogel concentrations (i.e., 10% *w*/*v* and 15% *w*/*v*), and then stirred and kept at 4 °C. After complete solubilization, the additional 20% of volume, represented by CB-PL, was added, and the samples were stirred again until homogeneous hydrogels were obtained. Formulations containing CB-PL will be referred to with the acronyms SHP407 10% *w*/*v*_PL 20% *v*/*v* and SHP407 15% *w*/*v*_PL 20% *v*/*v*.

#### 2.6.4. Effect of CB-PL Loading on Hydrogel Properties

The possible influence of CB-PL encapsulation on the hydrogel LCGT and gelation time at physiological temperature was investigated by the tube inverting test as previously described. The rheological characteristics of CB-PL-loaded gels were also studied as for pristine SHP407 hydrogels.

#### 2.6.5. Swelling and Stability of PL-Loaded Hydrogels During Incubation in Aqueous Media at 37 °C

The stability and swelling properties of CB-PL-loaded hydrogels in simulated biological environment (i.e., 37 °C, pH 7.4) were studied according to a previously developed protocol [52]. Tests were conducted on hydrogel samples (800 µL) prepared in Bijou containers as previously described. Before the beginning of the tests, all the samples were weighed (w_i_) and equilibrated at 37 °C for 15 min. Then, 800 µL of PBS equilibrated at 37 °C were added on each gel, and the samples were incubated for predefined time intervals (1, 3, 7, 10, and 14 days) at 37 °C. Three samples were prepared for each time point, and the medium was refreshed three times a week. At the end of each considered time point, the residual PBS was removed, and samples were weighed again (w_f_). To assess the loss of the polymeric/payload counterpart during incubation, each vial was also freeze-dried (Martin Christ ALPHA 2-4 LSC, Osterode am Harz, Germany) and weighed (w_f, dried_). As reference for the initial weight of the hydrogels, a set of samples was also freeze-dried and weighed without incubation with PBS (w_i, dried_). Finally, hydrogels’ swelling (i.e., PBS absorption) and stability (i.e., weight loss of the polymeric/payload counterpart) were calculated using Equations (1) and (2).

(1) $$swelling\;\left(\%\right)=\frac{\left(w_{f}-w_{i}\right)\cdot 100}{w_{f}}$$

(2) $$weight\;loss\;\left(\%\right)=\frac{\left(w_{i,\;dried}-w_{f,\;dried}\right)\cdot 100}{w_{i,\;dried}}$$

#### 2.6.6. Characterization of CB-PL Release Profile

The commercially available bicinchoninic acid (BCA) assay (Sigma Aldrich, Milano, Italy) was used to define the release profile of all the protein components of CB-PL from the formulated SHP407-based hydrogels. Firstly, the CB-PL-loaded hydrogel samples (800 µL) were prepared as described and incubated at 37 °C for 15 min to ensure complete gelation. Then, 800 µL of PBS equilibrated at 37 °C were added to each vial as release medium, and the samples were incubated again. After pre-determined time points (1, 3, and 5 h; 1, 2, 3, 7, 10, and 14 days), the PBS was taken from the vials and substituted with 800 µL of fresh medium. The collected solutions were then analyzed using the BCA assay kit according to the supplier’s instructions. The CB-PL solution as such was also diluted with PBS to obtain reference standards. Absorbance at 560 nm was measured with a Multimode Plate Reader VICTOR X3 (Perkin Elmer, Waltham, MA, USA) for both samples and standard solutions, from which a calibration curve was derived (linearity was found for standards with a concentration ranging from 1% *v*/*v* to 0.01% *v*/*v* with respect to the native CB-PL solution) and used to determine the final CB-PL concentration in the release media.

#### 2.6.7. Characterization of Platelet-Derived Growth Factor (PDGF) Release Profile

The release medium collected from CB-PL-loaded SHP407-based hydrogels at the predefined time points reported in the previous paragraph was also analyzed by the enzyme-linked immunosorbent assay (ELISA, Merck Millipore, Burlington, MA, USA) to quantify the levels of human platelet-derived growth factor-AB (PDGF-AB) in the eluates. The assay was performed according to the manufacturer’s instructions. The amount of PDGF-AB was determined from a calibration curve based on standards with a known PDGF-AB concentration. The cumulative release of PDGF-AB over a 14-day period was calculated by adding the release values at each time points and reported in mg.

### 2.7. In Vitro Biological Characterization

#### 2.7.1. Preparation of CB-PL-Loaded Hydrogels and Collection of Extracts for In Vitro Biological Evaluation

CB-PL-loaded SHP407-based hydrogels were prepared as described in Section 2.5.1 and Section 2.6.3. For the collection of hydrogel extracts for in vitro biological testing, 250 µL of hydrogel solution were placed in Bijou vials at 37 °C to allow gelation; then, 2.5 mL of serum-free medium were added, and the samples were incubated at 37 °C under static conditions. At 1, 2, 3, and 7 days, the gel extract was collected, and an equal volume of medium without serum was added.

#### 2.7.2. Cell Culture

The bioactivity of released CB-PL was evaluated through cell viability, proliferation, and migration assays using L929 murine fibroblasts and HaCat human keratinocytes. L929 (ICLC ATL95001, Biobanking and Cell Factory Hospital San Martino, Genova, Italy) and HaCat (BS CL 168, Istituto Zooprofilattico Sperimentale della Lombardia e dell’Emilia-Romagna “Bruno Ubertini”) were cultured in RPMI 1640 supplemented with 10% fetal bovine serum (FBS, Merck, Milano, Italy), 2 mM L-Glutamine, 100 μg/mL streptomycin, and 100 U/mL penicillin. The medium was routinely changed every 3 days, and at confluence, cells were subcultured (split ratio 1:3) by trypsinization (0.5% trypsin/0.02% EDTA). Cells were cultured at 37 °C in a humidified atmosphere with 5% CO_2_. Experiments aiming to evaluate CB-PL bioactivity were performed in the absence of serum to avoid a cytotoxic surplus of nutrients [55,56,57].

#### 2.7.3. Cell Viability Assay

Cell viability was tested using a tetrazolium-based colorimetric assay with MTT [3-(4,5 dimethylthiazol-2-yl)-2,5-diphenyl tetrazolium bromide] (Merck Life Science, Milano, Italy). Briefly, L929 (4 × 10^3^ cells/well) were seeded into 96-well plates. After 24 h of incubation, the medium was replaced with 200 μL/well of extracts. Complete medium, serum-free medium, and CB-PL 2% in serum-free medium were used as control samples. After 72 h of cell culture at 37 °C, 20 μL of an MTT solution (0.5 mg/mL) were added and incubated at 37 °C for 4 h. The supernatant was then removed from the wells and replaced with dimethyl sulfoxide (DMSO) (100 μL per well) to solubilize the MTT tetrazolium dye. The optical density (OD) was measured at 550 nm using a microplate reader (Spectrafluor Plus; TECAN Austria GmbH, Grödig, Austria). The percentage of cell viability was calculated vs. the complete medium control (assumed as 100%).

#### 2.7.4. Cell Proliferation Assay

Cell proliferation was evaluated by 5-bromo-2′-deoxyuridine (BrdU) incorporation assay (Roche Diagnostics, Mannheim, Germany). L929 (4 × 10^3^ cells/well) cell lines were seeded into 96-well plates. After 24 h of incubation, the medium was replaced with 200 μL/well of extracts. Complete medium, serum-free medium, and CB-PL 2% in serum-free medium were used as references. After 48 h of incubation, cell proliferation was assessed according to the manufacturer’s instructions. The OD was measured at a 450 nm wavelength using the microplate reader. The percentage of cell proliferation vs. the complete medium control sample was calculated (assumed as 100%).

#### 2.7.5. Cell Migration Assay

A scratch closure assay was performed to evaluate CB-PL potential in increasing the in vitro migration of keratinocytes on scratch-wounded monolayer models. HaCat (2 × 10^5^ cells/well) were seeded into 24-well plates, cultured to confluence, and scratched with a 10 μL pipette tip. Following PBS washes, cultures were re-fed with 500 μL/well of eluates. Control wells received CB-PL 2% serum-free medium or serum-free medium. Scratch closure was analyzed after 20 h of exposure to different time point eluates and control media. To this aim, digital images of cells were captured by a phase-contrast microscope (Axiovert 25, Zeiss, Milan, Italy; O.M. 50X) equipped with a digital camera (EOS 1000D, Canon, Milano, Italy).

### 2.8. Statistical Analysis

PEUs and hydrogel characterizations were performed in triplicate and results were reported as mean ± standard deviation. Statistical analysis was performed using the GraphPad^®^ Prism 5.0 Software (One-way ANOVA Calculator, Tukey HSD). In vitro tests were also performed in triplicate, and data were analyzed using StatViewTM 5.0 software (SAS Institute, Cary, NC, USA). The means were statistically compared using Student’s independent *t*-test. Values of *p* < 0.05 were considered statistically significant.

## 3. Results

### 3.1. PEU Chemical Characterization

#### 3.1.1. NHP407 Chemical Characterization

To assess the success of NHP407 synthesis, Attenuated Total Reflectance Fourier Transform Infrared Spectroscopy (ATR-FTIR) analyses were performed both on the PEU and on P407 as a comparison. The results are reported in Supplementary Figure S1. The NHP407 spectrum presents the specific absorption peaks of PEO-PPO-PEO triblock copolymers derived from the presence of P407 as a building block within the material. The peaks at 2876 and 1242 cm^−1^ can be ascribed to -CH_2_ stretching and rocking vibrations, respectively, while the peak at 1097 cm^−1^ can be attributed to -C-O-C- stretching vibration due to the repeated –OCH_2_CH_2_ PEO units in the macrodiol. The appearance of new absorption bands in the NHP407 spectrum compared to P407 proved the correct formation of urethane bonds, particularly the N-H (amide II) stretching vibration at 3347 cm^−1^, the stretching of free urethane carbonyl groups (amide I, C=O) at approximately 1722 cm^−1^, and the N-H (amide II) bending and C-N stretching vibrations at about 1530 cm^−1^. Finally, the absence of unreacted isocyanate groups was demonstrated by the absence of peaks at 2200 cm^−1^. Proton nuclear magnetic resonance (^1^H NMR) spectroscopy (Supplementary Figure S2) further proved the formation of a poly(ether urethane) containing P407, HDI, and N-Boc Serinol as building blocks. The signals at around 1.36 ppm and in the range of 1.52–1.60 ppm can be attributed to the methylene protons of the HDI-deriving block, while the signals in the region of 3.11–3.17 ppm can be ascribed to the methylene protons adjacent to the newly formed urethane bonds. The singlet at 1.19 ppm was produced by the resonance of the methyl protons of the PPO block, while the methylene protons of the PEO blocks gave a singlet at 3.74 ppm. Lastly, the methyl protons of Boc-caging groups were responsible for the singlet at 1.48 ppm.

The definition of the molecular weight distribution profile by SEC revealed a $\bar{M_{n}}$ value of 30.9 kDa with a polydispersity index of 1.8 (instrument error ± 10%) [58] in accordance with our previous results [52].

#### 3.1.2. SHP407 Chemical Characterization

To confirm that the acidic chloroform/TFA mixture used to expose –NH_2_ groups in SHP407 did not induce significant PEU degradation, the ATR-FTIR and ^1^H NMR spectra of SHP407 were recorded and compared to the NHP407 ones (Supplementary Figures S1 and S2). The two ATR-FTIR spectra (Supplementary Figure S1) appeared to be completely overlapped, thus confirming that the deprotection process preserved the polymer chain’s integrity. Similar conclusions were gathered from the comparison of the ^1^H NMR spectra of NHP407 and SHP407 (Supplementary Figure S2); the two spectra were completely overlapped and only differed in the signal at 1.48 ppm noting a chemical shift due to the methyl protons of Boc groups. Upon the acidic treatment leading to the removal of the Boc groups, the intensity of this signal significantly decreased in the SHP407 spectrum compared to the NHP407 one, proving the success of the Boc cleavage reaction and the achievement of a substantially complete Boc removal (i.e., 100% deprotection yield). These results were further confirmed by SEC analysis, with SHP407 giving results comparable to NHP407 ($\bar{M_{n}}$ = 31.4 kDa; polydispersity index = 1.7).

### 3.2. SHP407-Based Hydrogel Characterization

#### 3.2.1. Tube Inverting Test

The tube inverting test was performed to confirm the thermo-responsive behavior of SHP407-based hydrogels prepared at the two selected concentrations. As already established elsewhere [50], both the LCGT and gelation time at 37 °C were dependent on the hydrogel polymeric concentration; both parameters tended to decrease with increasing polymer concentration within the formulation. The SHP407 10% *w*/*v* and SHP407 15% *w*/*v* sol–gel systems exhibited LCGT values of 32 °C and 25 °C and gelation times of 8 min and 5 min at 37 °C, respectively. As an example, Supplementary Figure S3 reports representative images of a SHP407 15% *w*/*v* hydrogel in the sol (i.e., at 4 °C) and gel (i.e., at 37 °C) states.

The tube inverting test results made both the SHP407 10% *w*/*v* and SHP407 15% *w*/*v* hydrogel systems suitable for application in the biomedical field as they exhibited (i) a sol-to-gel transition between room temperature and physiological temperature, and (ii) a gelation time of a few minutes in physiological conditions (i.e., 37 °C) [51].

#### 3.2.2. Rheological Characterization

**Figure 1:**
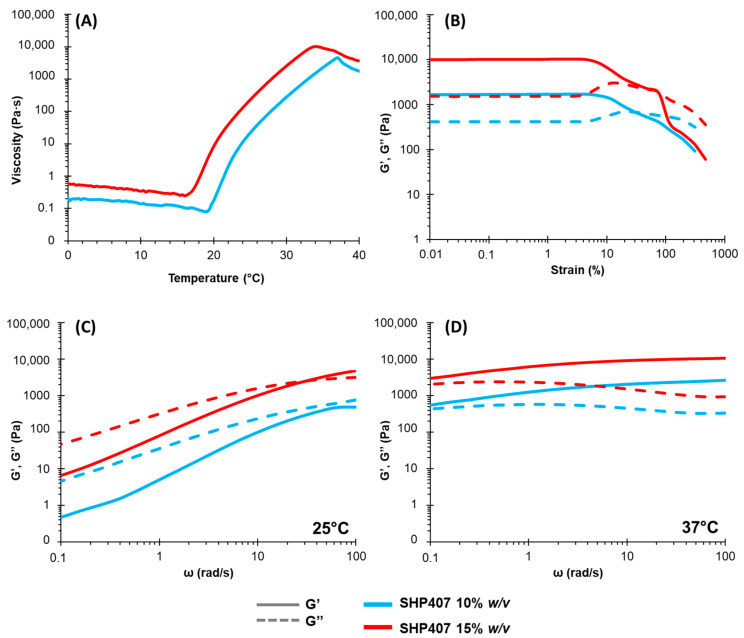
Rheological characterization of SHP407-based hydrogels with 10% and 15% *w*/*v* concentrations (in light blue and red, respectively): (**A**) trend of viscosity as function of temperature measured during temperature ramp test; (**B**) trends of storage (G′) and loss (G″) moduli (continuous and dashed lines, respectively) as function of applied deformation measured during strain sweep test at 37 °C; (**C**,**D**) G′ and G″ trends (continuous and dashed lines, respectively) as function of angular frequency measured during frequency sweep test at 25 and 37 °C.

Rheological tests were performed to quantitatively analyze the thermal response of the SHP407-based hydrogels. Temperature ramp tests were conducted to register the viscosity (η) vs. temperature trend in the temperature range of 0–40 °C (Figure 1A). As already observed elsewhere for analogous systems [51] and in accordance with the tube inverting test results, η was dependent on the system concentration. For both considered hydrogels, an initial decrease in viscosity was registered as being typical of fluid systems subjected to a temperature increase. Then, a steep increase in viscosity, corresponding to the beginning of the sol-to-gel transition, micelle formation, and progressive arrangement into an organized gel network, was detected, and the starting temperature associated with this phenomenon (corresponding to η inflection) was defined as T_onset_. Finally, instead of showing a plateau in the viscosity values when gelation was complete, both systems showed a melt fracture behavior. The T_onset_ values found for SHP407 10% *w*/*v* and SHP407 15% *w*/*v* (Table 1) were 19.03 and 16.03 °C, respectively. These values confirmed the expected dependency of both T_onset_ and η on the system concentration; by increasing the PEU content within the hydrogel, T_onset_ decreased, and the associated viscosity value increased. Figure 1B reports the results of the strain sweep tests performed at 37 °C on the two developed formulations. Again, the gel network’s ability to resist an applied deformation proved to be affected by the formulation’s polymeric content. The LVE region in which the storage (G′) and loss (G″) moduli showed stable values was evidenced for strain values below 11.6% for SHP407 10% *w*/*v* and 7.25% for SHP407 15% *w*/*v*. Within the LVE, the G′ values were always higher than those of G″ for both SHP407 10% *w*/*v* and SHP407 15% *w*/*v*, confirming that at 37 °C, the systems were in the gel state. Yield stress (YS) values, which were taken as the shear stress values at the maximum of G″, were found to be 171 and 641 Pa for SHP407 10% *w*/*v* and SHP407 15% *w*/*v*, respectively. At strain values between the LVE region and the maximum value of G″, G′ started to decrease due to progressive instability in the network (the formation of micro-cracks), while the subsequent decrease in G″ values indicated the formation of macro-cracks, ultimately leading to the prevalence of a viscous behavior over an elastic one (G″ > G′). In general, it was confirmed that hydrogels with a higher concentration were less resistant to applied deformation due to the higher rigidity of the formed network linked to their higher organization. Finally, frequency sweep tests at two different temperatures (25 and 37 °C) were performed to better characterize the gelation kinetics of the systems (Figure 1C). Comparing the two systems’ behavior at the same temperature, the effect of the polymer concentration was visible in characteristic parameters such as the G′ and G″ values at 100 rad/s and the angular frequency at the crossover between G′ and G″ (ω_G′/G″ crossover_), as reported in Table 1. At 25 °C, the SHP407 10% *w*/*v* sample behaved as a solution, with the G″ values being higher than the G′ ones within the angular frequency range between 0.1 and 100 rad/s. Differently, SHP407 15% *w*/*v* was a biphasic system; at an angular frequency higher than ω_G′/G″ crossover_ of 32.45 rad/s, the system behaved as a gel (G′ > G″), while at an angular frequency lower than 32.45 rad/s, the formulation showed the typical behavior of a solution. On the other hand, at 37 °C, ω_G′/G″ crossover_ was not visible in the frequency range analyzed, and G′ was higher than G″, signaling that both SHP407 10% *w*/*v* and SHP407 15% *w*/*v* were in the gel state. However, both G′ and G″ were still dependent on the angular frequency, which is typical of gels that are not fully developed yet, and a further increase in temperature would be needed to reach this condition.

Overall, the observed rheological properties further proved the suitability of the developed hydrogels for biomedical applications and, more specifically, for wound treatment: (i) the sol-to-gel transition occurs in a temperature range suitable for formulation handling and application in the wound site where the gel state will be achieved within a few minutes; (ii) the measured mechanical strength and resistance to applied deformation allow for hydrogel adaptation to the wound site and the capability to accommodate applied strain, avoiding stress application to the boundary tissues; and (iii) the trend of complex viscosity vs. angular frequency (data not reported) highlights a shear thinning behavior for both hydrogels, evidencing the capability of the formulations to decrease their viscosity upon shear stress application, thus enabling their injection.

### 3.3. Characterization of CB-PL-Loaded Hydrogels

#### 3.3.1. Effect of CB-PL Loading on Hydrogels Properties

The CB-PL-loaded PEU hydrogels were first characterized using the tube inverting test to primarily investigate the macroscopic effects of payload encapsulation on the gelation temperatures and gelation time at 37 °C. Irrespective of the SHP407 content, CB-PL loading at a final concentration of 20% *v*/*v* accelerated the gelation process. Formulations containing CB-PL, named SHP407 10% *w*/*v*_PL 20% *v*/*v* and SHP407 15% *w*/*v*_PL 20% *v*/*v*, showed LCGT values of 30 and 23 °C, respectively, compared to 32 °C for SHP407 10% *w*/*v* and 25 °C for SHP407 15% *w*/*v*. CB-PL loading also influenced the gelation time at 37 °C, which decreased from 8 min for SHP407 10% *w*/*v* to 7 min for SHP407 10% *w*/*v*_PL 20% *v*/*v* and from 5 min for SHP407 15% *w*/*v* to 4 min for SHP407 15% *w*/*v*_PL 20% *v*/*v*. The observed acceleration of the gelation process is in accordance with what was previously reported on PEU-based hydrogels with a similar composition and loaded with sodium dodecyl sulfate (SDS)-decorated mesoporous carbon particles [59] or poly(urethane)-coated silica nanoparticles [51]. Similarly to SDS molecules and poly(urethane) coating that most likely established non-covalent interactions with the hydrogel constituent PEU chains, the proteins contained in the CB-PL counterpart in the formulations developed in this study are believed to act as binding agents between the micelles formed by SHP407 via hydrogen bond formation, thus speeding up gel network formation.

**Figure 2:**
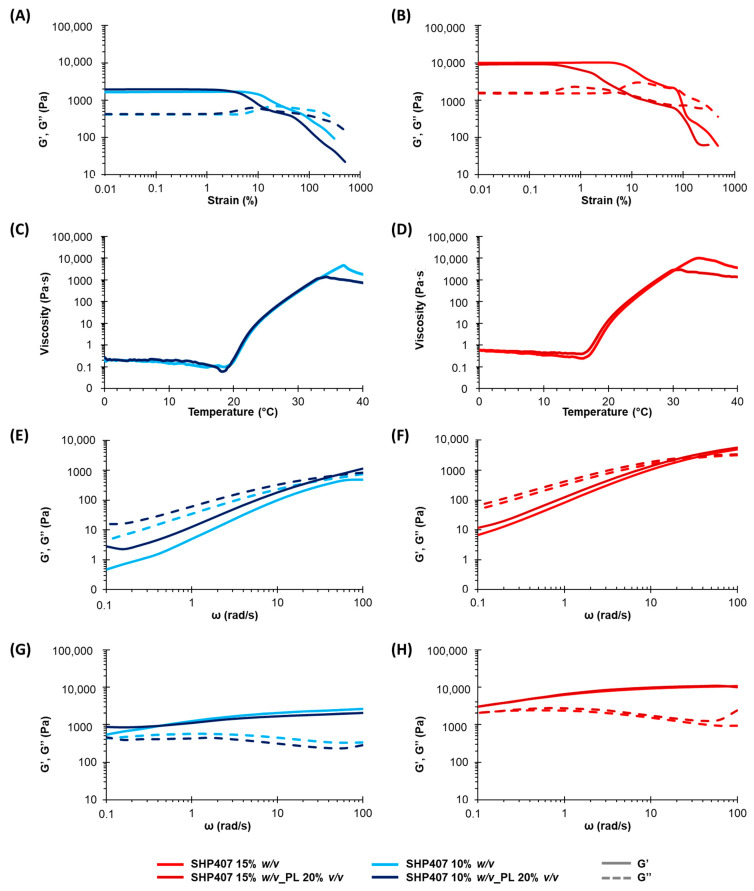
Rheological characterization of PL-loaded SHP407-based hydrogels. (**A**,**B**) Strain sweep tests at 37 °C. (**C**,**D**) Temperature ramp tests. (**E**,**F**) Frequency sweep tests at 25 °C. (**G**,**H**) Frequency sweep tests at 37 °C.

Rheological tests further confirmed these observations. Figure 2C,D report the trend of viscosity as a function of temperature measured for SHP407 10% *w*/*v*_PL 20% *v*/*v* and SHP407 15% *w*/*v*_PL 20% *v*/*v*, respectively, compared to their respective not-loaded hydrogels. For the CB-PL-loaded hydrogels, the derived T_onset_ values (Table 1) showed a slight decrease compared to pristine formulations, which is in agreement with the tube inverting test results (from 19.03 °C for SHP407 10% *w*/*v* to 18.37 °C for SHP407 10% *w*/*v*_PL 20% *v*/*v* and from 16.03 °C for SHP407 15% *w*/*v* to 15.70 °C for SHP407 15% *w*/*v*_PL 20% *v*/*v*), and melt fracture occurred at lower temperatures, suggesting the formation of stronger gel networks. The effect of CB-PL loading on the gelation kinetics was also detectable from frequency sweep tests, with a shift of ω_G′/G″ crossover_ towards lower values occurring when the payload was present (Table 1), indicating a faster sol-to-gel transition and a higher degree of network development both at 25 °C (Figure 2E,F) and at 37 °C (Figure 2G,H). However, in this case, the storage and loss moduli were also not independent of the angular frequency, demonstrating that even in the presence of CB-PL, the hydrogels did not achieve a fully developed gel state at 37 °C.

Regarding the strain sweep test results, on the other hand, the CB-PL effect was more marked: the presence of CB-PL constituent proteins is believed to introduce defects in the final gel network that could serve as a nucleation point for crack formation, leading to faster hydrogel failure when a deformation is applied. Indeed, the LVE region limit lowered from 11.60% for SHP407 10% *w*/*v* to 4.53% for SHP407 10% *w*/*v*_PL 20% *v*/*v* and from 7.25% for SHP407 15% *w*/*v* to 0.17% for SHP407 15% *w*/*v*_PL 20% *v*/*v* (Table 1), and the same effect was also visible for the YS values (Table 1).

#### 3.3.2. Swelling and Stability in Aqueous Media

**Figure 3:**
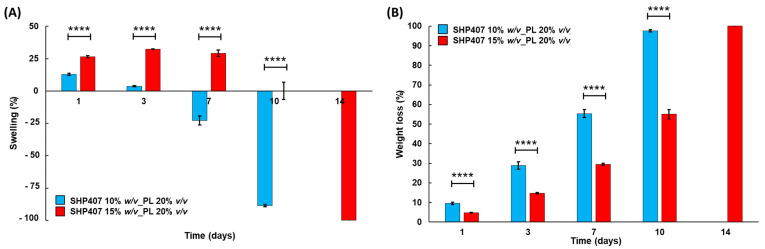
Stability tests for PL-loaded SHP407-based hydrogels. Percentages of (**A**) swelling and (**B**) dry weight loss measured for SHP407 10% *w*/*v*_PL 20% *v*/*v* (light blue) and SHP407 15% *w*/*v*_PL 20% *v*/*v* (red) samples over time. **** = *p* ≤ 0.0001.

The stability and swelling properties of the CB-PL-loaded hydrogels were evaluated by incubating the samples in contact with PBS at 37 °C. Figure 3 reports the percentages of swelling (Figure 3A) and dry weight loss (Figure 3B) measured at different time points for up to 10 days of observation. At each analyzed time point, SHP407 15% *w*/*v*_PL 20% *v*/*v* showed a significantly higher swelling percentage and lower weight loss compared to SHP407 10% *w*/*v*_PL 20% *v*/*v*. Indeed, it can be noted that for SHP407 15% *w*/*v*_PL 20% *v*/*v*, water absorption phenomena were prevalent in the first 72 h, with progressively increasing swelling percentages up to 32.2 ± 0.3%, and low weight loss of up to 14.7 ± 0.4%. After 7 days, on the other hand, dissolution phenomena started to dominate the hydrogel systems’ behavior, with a progressive decrease in the swelling percentage and a weight loss reaching 55.0 ± 2.3 % after 10 days of incubation. A similar behavior could be detected for the SHP407 10% *w*/*v*_PL 20% *v*/*v* samples, but in this case, dissolution phenomena were predominant from earlier incubation times. Indeed, the maximum swelling percentage (12.9 ± 0.8%) was reached after 24 h of incubation, and the percentage rapidly decreased at the following time points, showing negative values from 7 days. This behavior was also confirmed by the higher weight loss values measured for the SHP407 10% *w*/*v*_PL 20% *v*/*v* samples compared to the SHP407 15% *w*/*v*_PL 20% *v*/*v* samples after 7 days of incubation (55.4 ± 2.1% *vs*. 29.5 ± 0.7%). The complete dissolution of the SHP407 10% *w*/*v*_PL 20% *v*/*v* hydrogels was achieved after 10 days of incubation. This indicates a more rapid dissolution of the systems presenting a lower polymeric concentration, which is in accordance with what was already observed in a previous work on similar hydrogels [51]. Moreover, by comparing the measured swelling and weight loss trends with the ones reported in the aforementioned study, the presence of the payload did not seem to alter the stability of the systems in a relevant way despite the slight differences detected in terms of rheological properties and thermal response. Overall, the performed stability and swelling tests evidenced that the developed formulations show the capability to absorb fluids from the surrounding environment while undergoing progressive destabilization and potentially releasing an encapsulated payload. In wound treatment, swelling and stability properties play a pivotal role in determining dressing suitability for specific wound types, for instance, depending on the amount of exudate they produce. This aspect strictly influences the payload release kinetics, too. Based on these practical considerations, the designed hydrogels will need specific characterizations both in vitro in wound mimicking conditions (e.g., using exudate-simulating fluids and at a hydrogel/fluid volume ratio similar to the real situation) and in vivo.

#### 3.3.3. Characterization of CB-PL Release Profile

**Figure 4:**
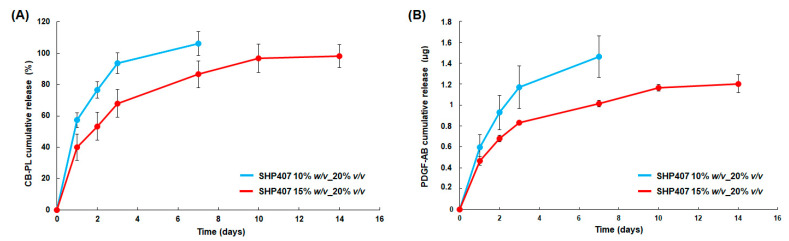
(**A**) CB-PL release profile (as percentages of the initial encapsulated amount) from the developed SHP407-based hydrogels measured through a BCA assay. (**B**) The PDGF-AB release profile obtained with the ELISA test.

CB-PL release was first estimated for both SHP407 10% *w*/*v*_PL 20% *v*/*v* and SHP407 15% *w*/*v*_PL 20% *v*/*v* using the BCA assay, which permitted us to evaluate the release of the whole payload protein components. The results are shown in Figure 4A. As expected from the stability test results described in the previous paragraph, the release kinetics was found to be dependent on the hydrogel polymeric concentration. Indeed, the SHP407 10% *w*/*v*_PL 20% *v*/*v* systems showed a faster payload release, which reached values of 93.7 ± 6.7% after 3 days and was complete after one week of incubation. On the other hand, the SHP407 15% *w*/*v*_PL 20% *v*/*v* systems presented a more sustained CB-PL release, with significantly lower values compared to the other formulation after 3 days of incubation (67.9 ± 8.8%, *p* ≤ 0.001). After one week of incubation, the payload was released at a percentage of 86.6 ± 8.5% and reached values of around 100% after 10 days. These results were in accordance with the higher stability of SHP407 15% *w*/*v*_PL 20% *v*/*v* systems: the payload release was better modulated in this case thanks to a later appearance of dissolution phenomena compared to formulations with a lower polymeric concentration. Hence, in agreement with previous evidence on similar formulations [52,54], the CB-PL release from the SHP407 10% *w*/*v*_PL 20% *v*/*v* and SHP407 15% *w*/*v*_PL 20% *v*/*v* systems was driven by a combination of diffusion and swelling/dissolution phenomena, with the latter being prevalent in SHP407 10% *w*/*v*_PL 20% *v*/*v* in accordance with the stability test results.

Similar results were also obtained from the estimation of PDGF-AB release from the two formulations (Figure 4B). PDGF-AB was selected as a target for a specific evaluation since it is considered one of the most relevant factors contained in platelet lysate for wound healing induction [27,60]. PDGF-AB release profiles were comparable to the whole protein content release trends estimated with the BCA assay, with more sustained release occurring over the considered two weeks’ timeframe for the SHP407 15% *w*/*v*_PL 20% *v*/*v* systems and a faster release for SHP407 10% *w*/*v*_PL 20% *v*/*v* due to an earlier occurrence of gel dissolution phenomena.

Considering the possible application of these sol–gel systems as topical drug delivery systems, the SHP407 15% *w*/*v*_PL 20% *v*/*v* hydrogel properties were considered more promising overall. Indeed, gelation occurred at lower temperatures (23 °C vs. 30°C for SHP407 10% *w*/*v*_PL 20% *v*/*v*) and in less time (4 vs. 7 min in physiological conditions), so easier placing and more efficient retaining on the positioning site could be envisioned. Additionally, the prolonged stability, improved rheological properties, and more controlled release of the encapsulated CB-PL shown by SHP407 15% *w*/*v*_PL 20% *v*/*v* could ensure a longer therapeutic effect of the patch. For these reasons, this formulation was selected to perform the subsequent in vitro tests to assess cytocompatibility and bioactivity.

### 3.4. In Vitro Biological Characterization of SHP407 15% w/v_PL 20% v/v

**Figure 5:**
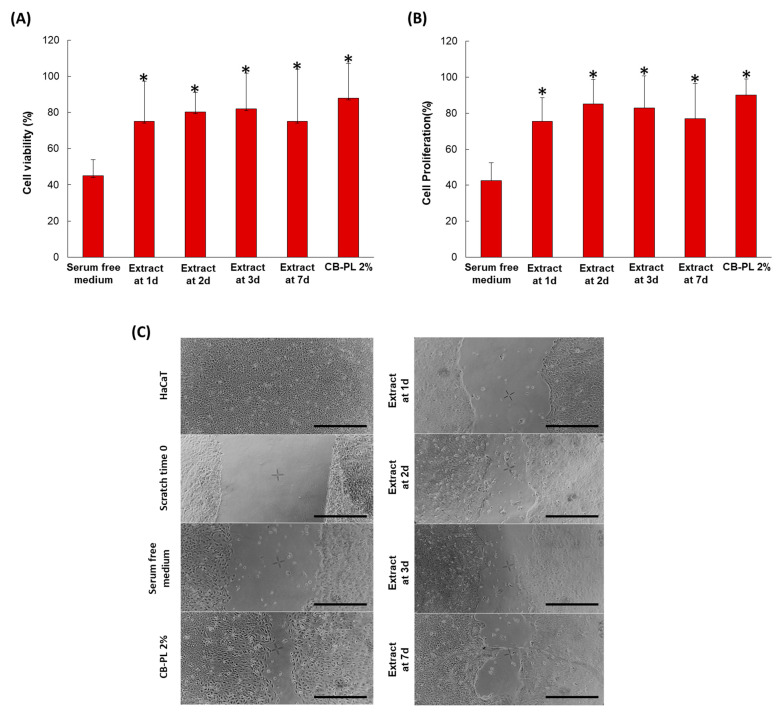
(**A**,**B**) Effects of released CB-PL from SHP407 15% *w*/*v*_PL 20% *v*/*v* gel on viability and proliferation of L929 cells. Viability (**A**) and proliferation (**B**) were assessed using MTT assay and BrdU incorporation assay, respectively, following 72 and 48 hours of incubation with CB-PL containing hydrogel extracts collected at different time points (1, 2, 3, and 7 days). Percentages of cell viability and proliferation were calculated vs. complete medium (assumed as 100%). Data are reported as mean ± SD of values obtained from three independent experiments with three replicates each; * *p* < 0.05 vs. serum-free medium. (**C**) Phase-contrast micrographs of scratch test. Scratch wound closure was evaluated after 20 h of exposure to CB-PL containing hydrogel extracts collected at different time points (1, 2, 3, and 7 days), CB-PL 2% serum-free medium and serum-free medium (positive and negative controls, respectively). Scale bar = 500 μm.

From the cell viability and proliferation tests, the CB-PL-loaded systems were able to satisfy the basic requirements of biocompatibility in clinical use. Indeed, compared to the serum-free medium which was used as a negative control, all of the SHP407 15% *w*/*v*_PL 20% *v*/*v* eluates collected at the different time points induced an increase in viability and proliferation rates of the L929 cell line, similar to what was observed with the positive control (CB-PL 2% in the serum-free medium) (Figure 5A,B). Furthermore, when compared to the serum-free medium control, all CB-PL-loaded-gel-derived eluates induced time-dependent increased rates of migration and closure of HaCaT cells (scratch closure assay) after 20 h of culture (20, 57, 61, and 69% at days 1, 2, 3, and 7, respectively) (Figure 5C). Scratch-wound recovery in keratinocytes induced by SHP407 15% *w*/*v*_PL 20% *v*/*v* eluates was also similar to what observed in the positive control CB-PL 2% in serum-free medium (about 80%) [61]. This positive influence could be attributed to the specific release detected through the ELISA test of PDGF-AB, which is known to be a promoter of cell proliferation and migration [62,63].

## 4. Conclusions

In this study, innovative sol–gel systems based on an *ad hoc* synthesized poly(ether urethane), named SHP407, were developed and loaded with cord blood-derived platelet lysate (CB-PL) for wound healing applications. Sol–gel systems at two different SHP407 concentrations (10% *w*/*v* and 15% *w*/*v*) were produced and showed acceptable thermal and rheological characteristics, making them suitable for use as topical delivery systems. The addition of CB-PL did not significantly influence the gelation properties of the hydrogels. Gel stability in aqueous media and the release kinetics of the encapsulated payload was influenced by the system concentration, with increased permanence and more sustained release for the formulation prepared at a higher PEU concentration (i.e., SHP207 15% *w*/*v*_PL 20% *v*/*v*). This formulation was thus subjected to an in vitro biological evaluation, which evidenced the efficacy of this hydrogel in improving L929 fibroblast cells’ viability and proliferation rates and providing proper stimulation to human keratinocytes to induce their migration in the presence of a wound.

Overall, the developed hydrogel formulations show potential for applications in localized wound treatment as an effective way to modulate the release of CB-PL and prolong its healing properties. Furthermore, their temperature-dependent gelation process enables easy application and perfect filling of the wound cavity irrespective of their geometrical features. Meanwhile, their physical nature is responsible for easy dressing removal through washing with aqueous solutions, resulting in increased patient comfort and the absence of damage to newly formed tissues. Additionally, the prolonged stability of the developed formulations coupled with sustained CB-PL release over time opens the door towards the possibility to decrease the frequency of dressing substitution (typically every 1 to 3 days [64]) with consequent improvement in patient compliance and a significant reduction in the economic burden on healthcare systems. Lastly, the highly tunable composition of the developed hydrogels could contribute to the personalized medicine field, allowing for a fine modulation of the formulation stability, swelling potential, payload content, and release kinetics based on the wound-specific features (e.g., wound type, exudative properties, and severity). This approach will make wound care treatments more effective, enabling their definitions based on specific patient needs and resulting in a remarkable increase in the wound management success rate. In this context, and towards translation to clinical practice, future works will focus on the evaluation of gel’s therapeutic efficacy in more relevant scenarios, both in vitro in bioengineered wound models and in vivo.

## Figures and Tables

**Table 1:** The measured rheological parameters for SHP407 10% *w*/*v*, SHP407 10% *w*/*v*_PL 20% *v*/*v*, SHP407 15% *w*/*v*, and SHP407 15% *w*/*v*_PL 20% *v*/*v*. The table reports the following parameters: viscosity at 0 and 25 °C (η_0°C_ and η_25°C_), the onset temperature of the gelation process (T_onset_), the linear viscoelastic (LVE) region limit and yield stress (YS) at 37 °C, the angular frequency at the crossover between G′ and G″ (ω_G′/G″ crossover_), and the G′ and G″ values at 100 rad/s at 25 and 37 °C.

		SHP407 10% *w*/*v*	SHP407 10% *w*/*v* PL 20% *v*/*v*	SHP407 15% *w*/*v*	SHP407 15% *w*/*v* PL 20% *v*/*v*
η_0°C_ (Pa·s)		0.21	0.27	0.60	0.61
T_onset_ (°C)		19.03	18.37	16.03	15.70
η_25°C_ (Pa·s)		39.96	44.34	398.90	500.60
LVE (%)		11.60	4.53	7.25	0.17
YS (Pa)		217.00	87.90	770.00	52.20
ω_G′/G″ crossover_ (rad/s)	25 °C	x	51.45	32.45	20.45
	37 °C	<0.1	<0.1	<0.1	<0.1
G′–G″ 100 rad/s (Pa)	25 °C	487–758	1140–830	4940–3210	5720–3370
	37 °C	2650–336	2070–283	10,900–935	9990–2400

## Data Availability

The data presented in this study are available upon request from the corresponding author.

## Supplementary Materials

The following supporting information can be downloaded at https://www.mdpi.com/article/10.3390/pharmaceutics16111438/s1, Figure S1: ATR-FTIR spectra of P407 (black), NHP407 (red) and SHP407 (green); Figure S2: 1H NMR spectra of NHP407 (red) and SHP407 (green); Figure S3: Representative images of the temperature-driven sol-to-gel transition of a SHP407-based hydrogel with a 15% *w*/*v* concentration.
