# Supplementary material for: Cord Blood Platelet Lysate-Loaded Thermo-Sensitive Hydrogels for Potential Treatment of Chronic Skin Wounds
Source: Pharmaceutics. 2024 Nov 11;16(11):1438. doi: 10.3390/pharmaceutics16111438 (PMC11597581; doi:10.3390/pharmaceutics16111438)
Supplement: Supplementary file 1 [file pharmaceutics-16-01438-s001.zip › pharmaceutics-3264540-supplementary.pdf]

Cord Blood Platelet Lysate-Loaded Thermo-Sensitive Hydrogels for Potential Treatment of Chronic Skin Wounds

Arianna Grivet-Brancot [1,2], Marianna Buscemi [3], Gianluca Ciardelli [1,2,*], Simona Bronco [1], Susanna Sartori [2], Claudio Cassino [4], Tamer Al Kayal [3], Paola Losi [3], Giorgio Soldani [3] and Monica Boffito [1,2,*]

1 Institute for Chemical-Physical Processes, National Research Council, 56124 Pisa, Italy; arianna.grivet@polito.it (A.G.-B.); simona.bronco@pi.ipcf.cnr.it (S.B.)
2 Department of Mechanical and Aerospace Engineering, Politecnico di Torino, 10129 Torino, Italy; susanna.sartori@polito.it
3 Institute of Clinical Physiology, National Research Council, Massa, 56124 Pisa, Italy; maribuscemi@libero.it (M.B.); tamer.alkayal@ifc.cnr.it (T.A.K.); paola.losi@cnr.it (P.L.); giorgio.soldani@ifc.cnr.it (G.S.)
4 Department of Science and Technological Innovation, Università del Piemonte Orientale, 15121 Alessandria, Italy; claudio.cassino@uniupo.it
* Correspondence: gianluca.ciardelli@polito.it (G.C.); monica.boffito@polito.it (M.B.)

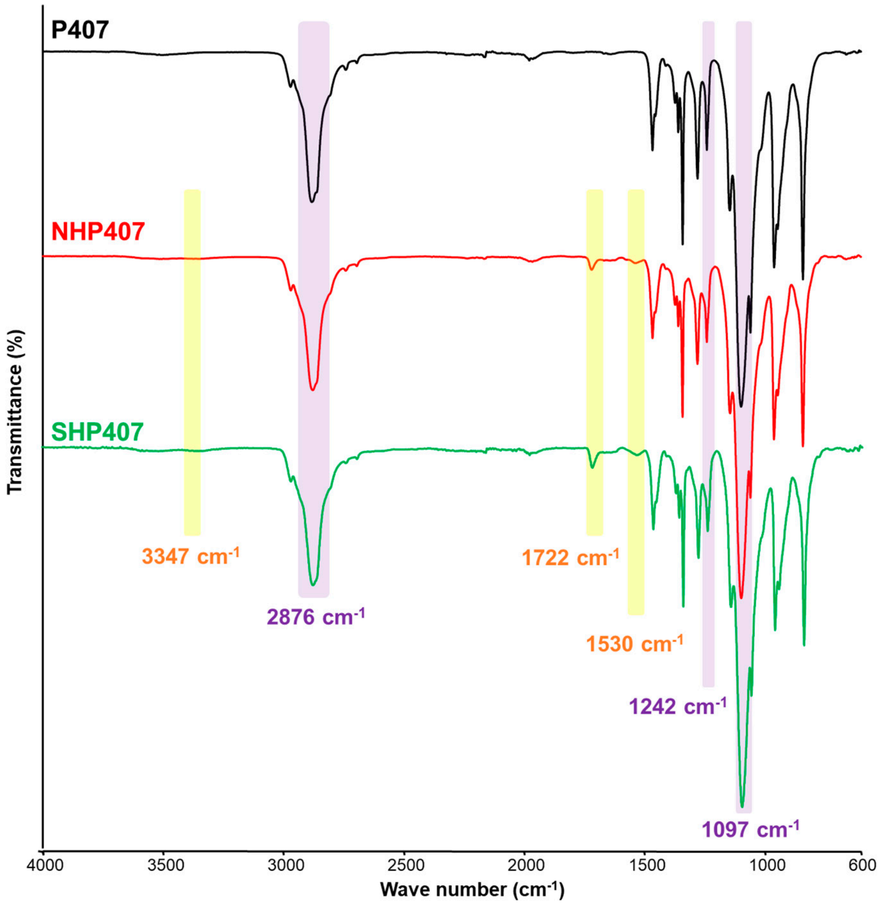

Figure S1. ATR-FTIR spectra of P407 (black), NHP407 (red) and SHP407 (green). Yellow and violet bars identify the characteristic peaks of newly formed urethane domains and P407 building blocks, respectively.

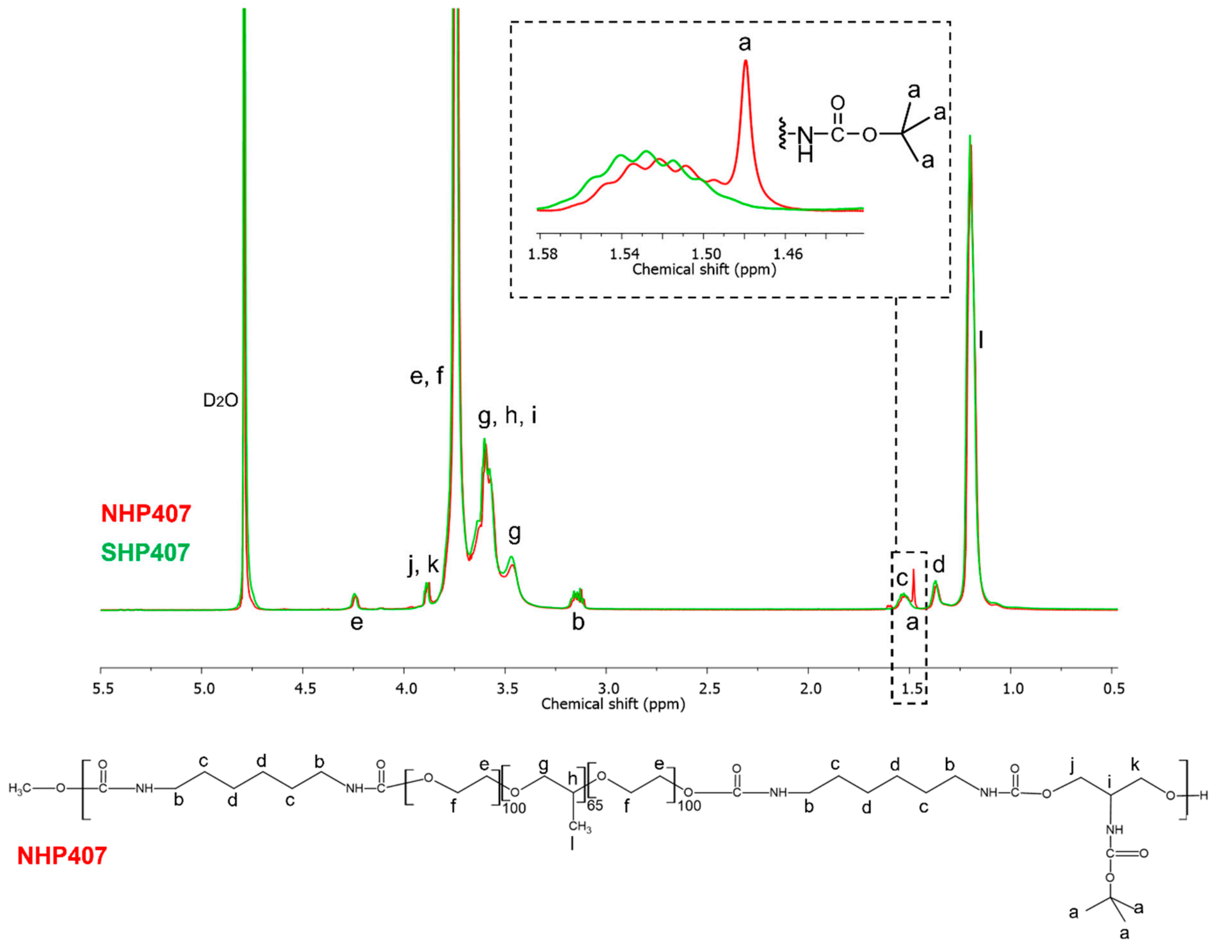

Figure S2. ^{1}H NMR spectra of NHP407 (red) and SHP407 (green). The magnified insert highlights the differences between the spectra induced by the Boc-cleavage reaction (i.e., disappearance of the peak at 1.48 ppm due to the methyl protons of Boc groups) leading to the synthesis of SHP407 starting from NHP407.

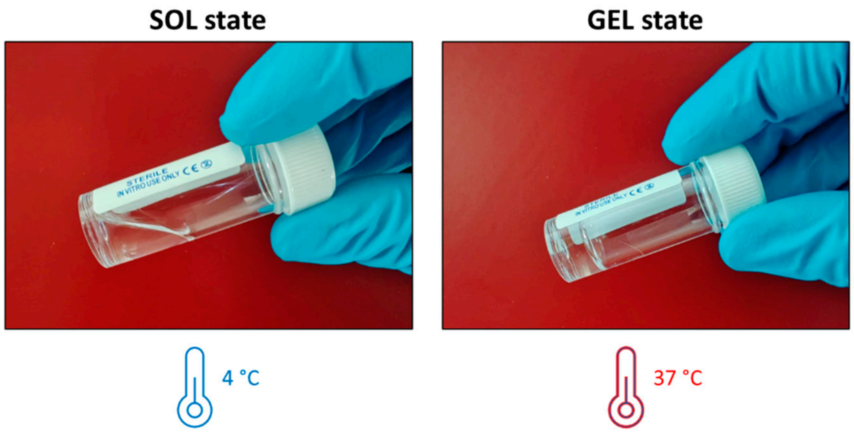

Figure S3. Representative images of the temperature-driven sol-to-gel transition of a SHP407-based hydrogel with 15% *w/v* concentration: sol (left) and gel (right) states at 4 °C and 37 °C, respectively.
